# Endurance performance and energy metabolism during exercise in mice with a muscle-specific defect in the control of branched-chain amino acid catabolism

**DOI:** 10.1371/journal.pone.0180989

**Published:** 2017-07-18

**Authors:** Minjun Xu, Yasuyuki Kitaura, Takuya Ishikawa, Yoshihiro Kadota, Chihaya Terai, Daichi Shindo, Takashi Morioka, Miki Ota, Yukako Morishita, Kengo Ishihara, Yoshiharu Shimomura

**Affiliations:** 1 Department of Applied Molecular Biosciences, Graduate School of Bioagricultural Sciences, Nagoya University, Nagoya, Japan; 2 Faculty of Agriculture, Ryukoku University, Fushimi-ku, Kyoto, Japan; University of Birmingham, UNITED KINGDOM

## Abstract

It is known that the catabolism of branched-chain amino acids (BCAAs) in skeletal muscle is suppressed under normal and sedentary conditions but is promoted by exercise. BCAA catabolism in muscle tissues is regulated by the branched-chain α-keto acid (BCKA) dehydrogenase complex, which is inactivated by phosphorylation by BCKA dehydrogenase kinase (BDK). In the present study, we used muscle-specific BDK deficient mice (BDK-mKO mice) to examine the effect of uncontrolled BCAA catabolism on endurance exercise performance and skeletal muscle energy metabolism. Untrained control and BDK-mKO mice showed the same performance; however, the endurance performance enhanced by 2 weeks of running training was somewhat, but significantly less in BDK-mKO mice than in control mice. Skeletal muscle of BDK-mKO mice had low levels of glycogen. Metabolome analysis showed that BCAA catabolism was greatly enhanced in the muscle of BDK-mKO mice and produced branched-chain acyl-carnitine, which induced perturbation of energy metabolism in the muscle. These results suggest that the tight regulation of BCAA catabolism in muscles is important for homeostasis of muscle energy metabolism and, at least in part, for adaptation to exercise training.

## Introduction

The branched-chain amino acids (BCAAs), leucine, isoleucine, and valine, are essential amino acids (EAAs) for mammals and have unique characteristics in their catabolic system; BCAAs are directly catabolized in skeletal muscle, whereas the other EAAs are mainly catabolized in liver [1]. This organ specificity of BCAA catabolism is attributed to the expression and activities of the first two enzymes, which are common to the three BCAAs, in the catabolic pathway. The first enzyme is a branched-chain aminotransferase (BCAT), which catalyzes transamination of BCAAs to produce corresponding branched-chain α-keto acids (BCKAs). The BCAT activity is relatively high in skeletal muscle, but the enzyme is almost not expressed in liver [2]. The second enzyme is BCKA dehydrogenase complex (BCKDC), which catalyzes the oxidative decarboxylation of BCKAs to produce corresponding CoA esters [1]. In contrast to BCAT, the activity of BCKDC is quite low in skeletal muscle, but extremely high in liver of rat and mouse [3, 4]. Therefore, the oxidation of BCAAs in skeletal muscle is regulated by BCKDC.

BCKDC is regulated by covalent modification; the enzyme is inactivated by phosphorylation of the E1α component of the complex and is activated by dephosphorylation [4]. The specific kinase (BCKA dehydrogenase kinase (BDK)) [5, 6] and phosphatase (BCKA dehydrogenase phosphatase (BDP)) [7, 8] are responsible for the reactions, respectively. It has been reported that BDK activity in rat skeletal muscle is very high compared to that in liver [9], contributing to the very low activity state of BCKDC (a low percentage of the complex in the dephosphorylated state) in skeletal muscle of rats under normal and resting conditions [3].

Endurance exercise enhances energy expenditure and promotes the oxidation of protein and amino acid catabolism. BCAA oxidation is enhanced by exercise in association with an increase in the BCKDC activity in human and rat skeletal muscle [10, 11] and rat liver [12]. Therefore, effects on exercise performance of acute BCAA supplementation before and during exercise and of chronic BCAA supplementation during the exercise training period have been examined. The results suggest that the effects of BCAAs were not clear with acute supplementation but were positive with chronic supplementation, which improved endurance performance likely through enhancing protein synthesis and mitochondrial biogenesis in humans and mice [13–15]. Furthermore, a recent study has investigated fuel selection in rats selectively bred for high and low intrinsic running capacity, and the results showed that the former animals used fatty acids and BCAAs as an energy source more efficiently than the latter [16]. However, no research has been conducted to examine the effects of promoted BCAA catabolism and BCAA deficiency in muscle tissues on exercise performance.

In the present study, we produced mice with a muscle-specific defect in the control of BCAA catabolism by muscle-specific knockout of the BDK gene [17]. As described above, the high BDK activity in muscle tissues suppresses BCAA oxidation; however, the mice with muscle-specific BDK defect (BDK-mKO mice) had high muscle BCKDC activity [17], resulting in promotion of BCAA oxidation. We examined endurance performance of the mice and conducted a metabolome analysis using the skeletal muscle of the mice during exercise.

## Materials and methods

### Ethics statement

All procedures were approved by the Animal Care Committee of the Nagoya University Graduate School of Bioagricultural Sciences.

### Animals

We generated BDK-mKO mice using the Cre-loxP system [18], as described previously [17]. In brief, BDK^flox/flox^ mice were produced using BDK-floxed (Neo+) ES cells, which had the targeting vector for the BDK allele with a frt-flanked LacZ/neo cassette integrated upstream of exon 9 and the floxed exons 9–12. The mice were mated with transgenic mice that had the Cre recombinase gene driven by a muscle creatine kinase promoter (#006475, Jackson Laboratory) to generate BDK-mKO (BDK^flox/flox^; Ckmm-Cre (+)) mice. Control mice (BDK^flox/flox^; Ckmm-Cre (-) mice) have been described previously [17]. These mice were housed at 23 ± 1°C with lights on from 08:00 h to 20:00 h. At 4 weeks of age, male mice were weaned and housed individually with free access to water and food (standard rodent chow CE-2, CLEA Japan, Tokyo).

### Exercise protocol

From 8 weeks of age, male control and BDK-mKO mice (n = 7–8 per group) were ad libitum fed an experimental diet (20 kcal% from protein, 70 kcal% from carbohydrate and 10 kcal% from fat; D12450B, Research Diets, Inc., New Brunswick, NJ, USA) until the end of the experiment. At the end of 11 weeks of age, the endurance capacity of all of the mice was assessed by measuring the distance of running to exhaustion using a motor-driven treadmill (Natsume, Tokyo, Japan) with a 10% grade. The running speed of the test was an initial 15 m/min for 4 min, which was then increased at a rate of 1 m/min every 4 min. Then, all of the mice were trained for 2 weeks (until 14 weeks of age) by running on the treadmill with a 10% grade for 60 min/day at a speed of 15 m/min in the initial 1 week and 18 m/min in the following 1 week, 5 days/week. The endurance capacity of all of the mice was assessed again using the above method 2 days after the last running training.

The same set of control and BDK-mKO mice (n = 9–10 per group) were prepared for the metabolome analysis. Both groups of mice were raised until 14 weeks of age with the running training for the final 2 weeks, as described above. On the final day of the experiment (2 days after the last running training), mice were deprived of food for ~8 h before sacrifice. Each group of mice was randomly divided into 2 subgroups: running and sedentary. The mice in the running subgroup were run on the treadmill with a 10% grade for 32 minutes at an initial speed of 15 m/min, which was increased at a rate of 1 m/min every 4 min (final speed at 23 m/min). This exercise bout was conducted between 15:00 and 17:00. Immediately after running, the mice were killed by cervical dislocation, after which the skeletal muscles (gastrocnemius and plantaris muscles) of the right hind-limb were rapidly removed, freeze-clamped at liquid nitrogen temperature, and then stored for the metabolome analysis. The soleus muscle of the right hind-limb was removed, freeze-clamped at liquid nitrogen temperature, and then stored for the enzyme activity analysis. From the left hind-limb, the soleus, gastrocnemius, and plantaris muscles were removed together, freeze-clamped at liquid nitrogen temperature, and stored at –80°C until use. Blood was collected from the posterior vena cava and/or heart to prepare plasma, and then heart, liver, kidney, pancreas, testis, and brain were removed, freeze-clamped at liquid nitrogen temperature, and stored at –80°C until used in analyses. Following the running subgroup, the mice in the sedentary subgroup were killed without running and treated using the same procedures as above.

The assay system for endurance exercise capacity was developed using a current swimming pool for mice [19]. We prepared the other set of control and BDK-mKO mice (n = 7–8 per group) to examine the endurance capacity using this assay system. Both groups of mice were raised until 12 weeks of age as described above, and then the mice were accustomed to swimming for 10 min/day for 2 days. Four days after the swimming practice, swimming endurance capacity was evaluated once a day for 4 consecutive days, as reported previously [20].

### Metabolite extraction and metabolome analysis

Metabolite extraction and metabolome analysis were conducted at Human Metabolome Technologies (HMT, Tsuruoka, Yamagata, Japan). Briefly, approximately 50 mg of frozen tissue were plunged into 1,500 μL of 50% acetonitrile/Milli-Q water containing internal standards (Solution ID: 304–1002; HMT) at 0°C in order to inactivate enzymes. The tissue was homogenized thrice at 1,500 rpm for 120 sec using a tissue homogenizer (Micro Smash MS100R; Tomy Digital Biology Co., Ltd., Tokyo, Japan), and the homogenate was then centrifuged at 2,300×g and 4°C for 5 min. Subsequently, 800 μL of the upper aqueous layer were centrifugally filtered through a Millipore 5-kDa cutoff filter at 9,100×g and 4°C for 120 min to remove proteins. The filtrate was centrifugally concentrated and re-suspended in 50 μL of Milli-Q water for CE-MS analysis at HMT.

Metabolome analysis was conducted using the Basic Scan package of HMT with capillary electrophoresis time-of-flight mass spectrometry (CE-TOFMS) based on the methods described previously [21, 22]. Briefly, CE-TOFMS analysis was carried out using an Agilent CE capillary electrophoresis system equipped with an Agilent 6210 time-of-flight mass spectrometer, Agilent 1100 isocratic HPLC pump, Agilent G1603A CE-MS adapter kit, and Agilent G1607A CE-ESI-MS sprayer kit (Agilent Technologies, Waldbronn, Germany). The systems were controlled by Agilent G2201AA ChemStation software version B.03.01 for CE (Agilent Technologies) and connected by a fused silica capillary (50μm i.d. ×80cm total length) with commercial electrophoresis buffer (H3301-1001 and H3302-1021 for cation and anion analyses, respectively; HMT) as the electrolyte. The spectrometer was scanned from m/z 50 to 1,000 [21]. Peaks were extracted using MasterHands, an automatic integration software (Keio University, Tsuruoka, Yamagata, Japan) in order to obtain peak information including m/z, peak area, and migration time (MT) [23]. Signal peaks corresponding to isotopomers, adduct ions, and other product ions of known metabolites were excluded, and the remaining peaks were annotated according to the HMT metabolite database based on their m/z values with the MTs. Areas of the annotated peaks were then normalized based on internal standard levels and sample amounts in order to obtain relative levels of each metabolite. The below-detection limit data were assigned as one-half the detection limit value for the statistical treatments [24].

### Western blotting

Antisera against BDK [9] and BCKDC [5] were prepared as reported previously. The protein concentration of the tissue extracts was adjusted to 5 μg protein/μL. Protein concentrations were determined using the bicinchoninic acid protein assay (Thermo Fisher Scientific, Waltham, MA, USA). SDS-PAGE was carried out on a 10% polyacrylamide separating gel at a constant current of 30 mA per gel for ∼1 h. The tissue extracts at 25–50 μg protein were loaded on each lane. After SDS-PAGE, the proteins were transferred to polyvinylidene fluoride (PVDF) membranes by using trans-blot cell (Bio-Rad, Hercules, CA, USA) at a constant voltage of 8 V for 2 h. After the transfer of protein, the membranes were blocked in blocking buffer (5% (w/v) skim milk in Tris-buffered saline (TBS) containing 0.1% (w/v) Tween 20 (TBST)) for 1 h. The membranes were incubated overnight at 4°C with polyclonal-BDK antibody diluted in blocking buffer. Blots were then incubated with rabbit IgG antibody for 2 h. Target immunoreactive proteins on the membranes were visualized using ECL Western blotting detection reagents (GE Healthcare UK Ltd., Buckinghamshire, UK). The exposure time was set to 1 min, since the changes in staining intensity were linear depending on the amount of the target protein. Next, we stripped the blots with stripping buffer (62.5 mM Tris-HCl (pH 6.7 at 20°C), 100 mM 2-mercaptoethanol, 2% (w/v) SDS) at 68°C for 45 min. After stripping, the membranes were reblocked with blocking buffer, incubated with BCKDC E2 antibody in blocking buffer, and then the steps described above were repeated.

### Determination of muscle glycogen

Muscle glycogen in the hind-limb muscle was determined by the phenol-sulfuric acid method, as reported previously [25].

### Assay of mitochondrial enzyme activities

Frozen soleus muscle was homogenized in extraction buffer containing 50 mM HEPES (pH 7.4 with NaOH), 1% (w/v) Triton X-100, 1 mM EDTA, 1 mM EGTA, 2 mM Na_3_VO_4_, 100 mM NaF, 50 mM Na_4_P_2_O_7_, 1 mM phenylmethylsulfonyl fluoride (PMSF), 0.02 mg/mL leupeptin, 5 μg/mL aprotinin, 0.1 mg/mL trypsin inhibitor, and 0.1 mM N-tosyl-L-phenylalanine chloromethyl ketone (TPCK) using a Digital Homogenizer (HOM; AS ONE Corporation, Osaka, Japan) with a Teflon pestle at a setting of 5 for 5 min on ice. The homogenate was centrifuged at 7000 rpm for 15 min at 4°C to obtain the supernatant. The supernatant was then used for analyses of mitochondrial enzyme activities without freezing.

The activity of citrate synthase in the supernatant was measured spectrophotometrically at 412 nm at 30°C [26]. The supernatant was added to the assay cocktail (final 1 mL) containing 0.1 M Tris-HCl buffer (pH 8.1), 0.1 mM 5,5'-dithiobis(2-nitrobenzoic acid) (DTNB), 0.3 mM acetyl-CoA, and 0.5 mM oxaloacetate. One unit of citrate synthase activity refers to the formation of 1 nmol of citrate/min.

The activity of cytochrome c oxidase in the supernatant was measured spectrophotometrically at 550 nm and 30°C [27]. The supernatant was added to the assay cocktail (final 1 mL) containing 10 mM sodium phosphate (pH 7.0), and 0.3 mg/mL cytochrome c. One unit of cytochrome c oxidase activity refers to the formation of 1 nmol of oxidized cytochrome c/min.

### Amino acid analysis

Plasma samples were mixed with 3% (w/v) sulfosalicylic acid to give a final concentration of 1.5% and then centrifuged at 3000 rpm at 4°C for 15 min to remove precipitated proteins. The amino acid concentrations in the supernatants obtained were analyzed using an automatic amino acid analyzer (JLC-500/V; JEOL, Tokyo, Japan).

### Statistical analysis

Data are expressed as means ± SEM. Analysis of the metabolome data was performed by HMT using Welch’s t-test. The data of exercise performance were compared between control and BDK-mKO mice using a Student’s t-test, and the other data were analyzed by the Tukey-Kramer method for multiple comparisons using StatView (version 5.0) software (SAS Institute, Cary, NC, USA). Probability of less than 0.05 was considered to be statistically significant.

## Results

### Exercise performance

**Fig 1:**
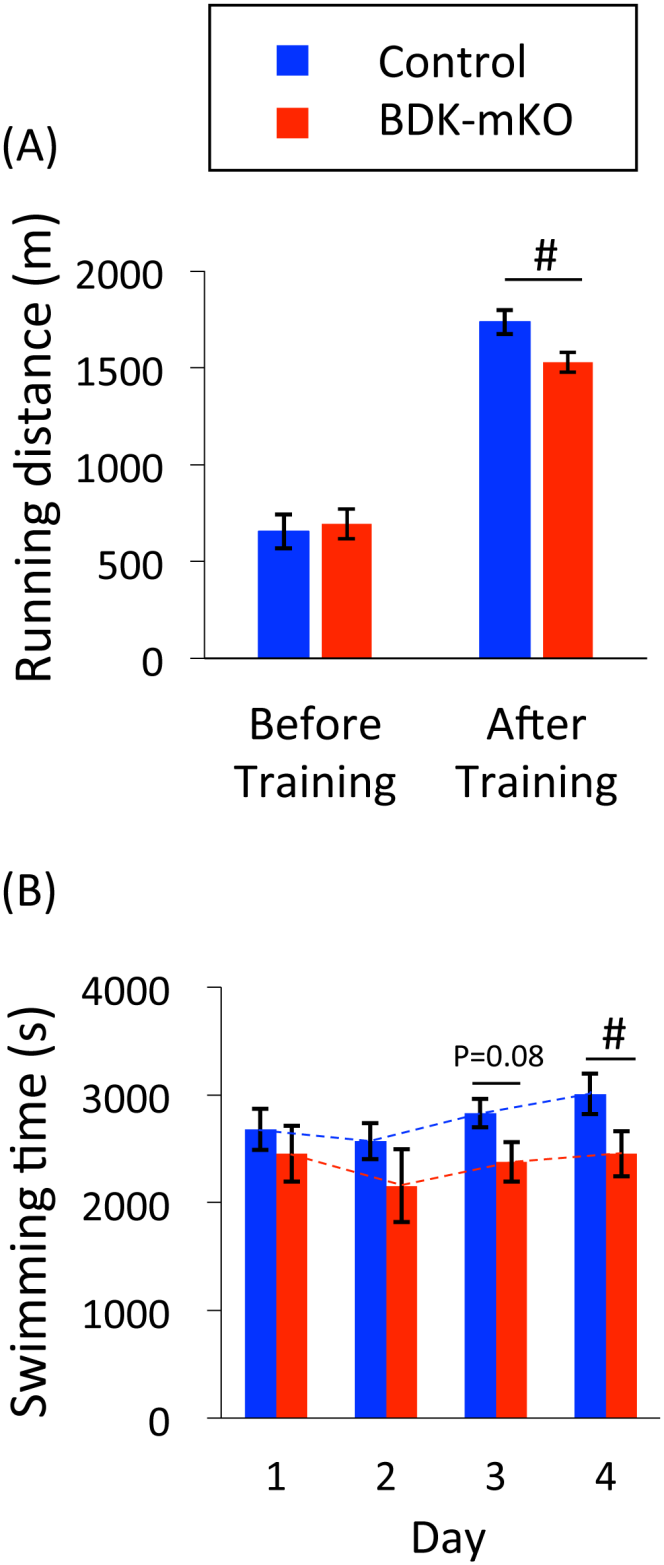
Exercise performance of control and BDK-mKO mice. **(A)** Running distance to exhaustion before and after 2 weeks of training, and (B) swimming time to exhaustion of untrained mice on each of 4 consecutive days. # Significant difference between control and BDK-mKO mice.

Running distance to exhaustion before the exercise training was not different between the control and BDK-mKO mice (Fig 1A). After 2 weeks of training, both groups of mice ran more than 2 times greater distance than that before training; however, the running distance was significantly less in BDK-mKO mice than in control mice (1528 ± 51 m vs. 1737 ± 62 m, respectively) (Fig 1A). In order to examine the adaptability of both mouse groups to exercise training, both groups of untrained mice were applied to swimming exercise. The swimming time to exhaustion was measured once a day for 4 consecutive days. The results showed that the swimming time tended to be increased in control mice, but not in BDK-mKO mice, resulting in significantly less swimming time in BDK-mKO mice than in control mice on day 4 (Fig 1B).

### Characterization of mice used in the metabolome analysis on the final experimental day

All of the control and BDK-mKO mice were trained for 2 weeks, as described in the Materials and Methods. One-half of the control and BDK-mKO mice were run for 32 min before being sacrificed, while the other half were sacrificed under the sedentary condition. No differences were observed in body and tissue weights on the final experimental day among the 4 groups of mice (S1 Table).

In BDK-mKO mice, BDK was not detected in heart and skeletal muscle, but was detected in other tissues examined (S1 Fig), as reported previously [17]. The amounts of the subunits of muscle BCKDC were not affected by exercise training in either control and BDK-mKO mice (S2 Fig).

**Fig 2:**
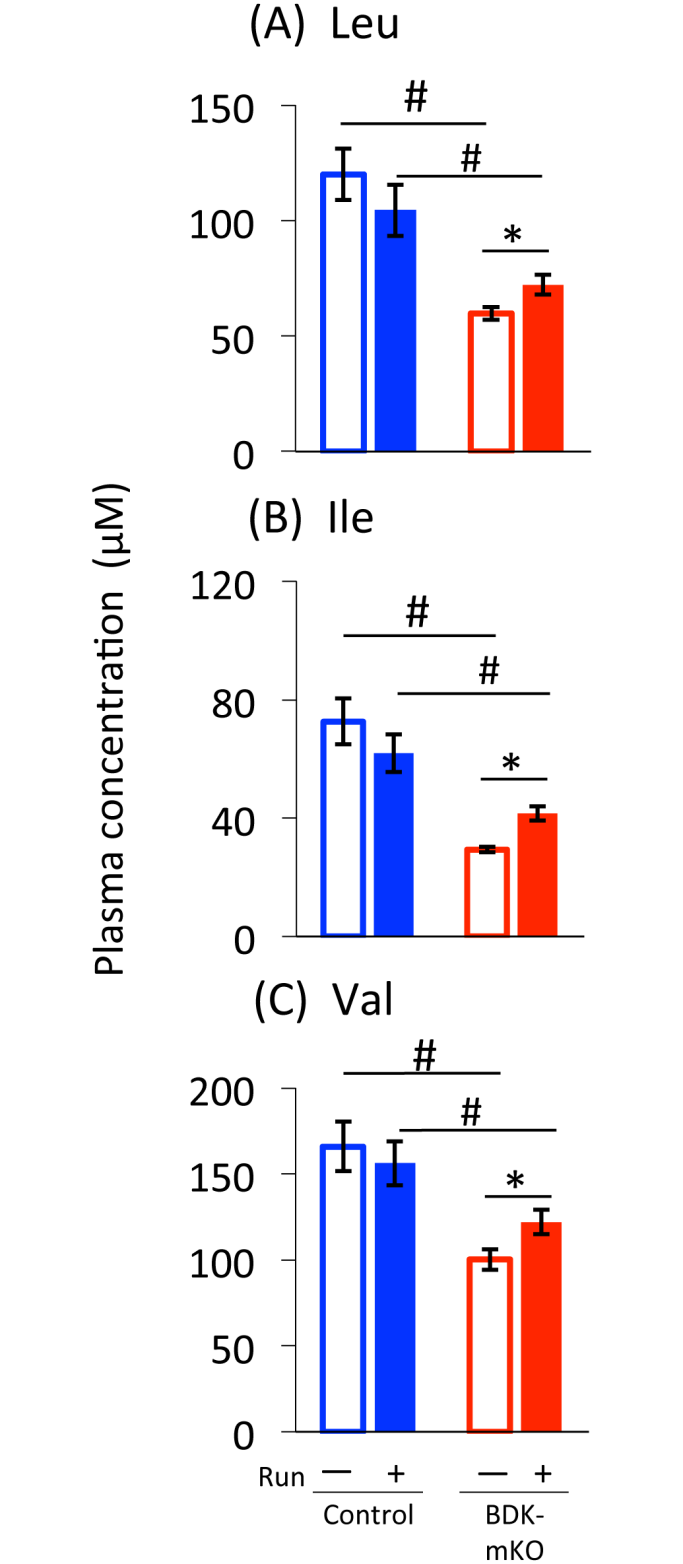
Plasma BCAA concentrations of control and BDK-mKO mice with and without the running exercise bout. # Significant difference between control and BDK-mKO mice. * Significant difference in the same group of mice with and without the exercise bout.

The concentrations of the three plasma BCAAs were significantly lower in the sedentary BDK-mKO mice than in the sedentary control mice (Fig 2). These concentrations tended to be decreased after the exercise bout in control mice, but the opposite was observed in BDK-mKO mice (Fig 2). The increases in the plasma BCAA concentrations in BDK-mKO mice might be related to the increases in the amino acid concentrations in tissues other than muscles such as liver [28]. The concentrations of other plasma amino acids (S3 Fig) were not different between the sedentary control and BDK-mKO mice, except that the aspartate concentration tended to be higher in BDK-mKO than in control. As observed for the concentrations of plasma BCAAs, the concentrations of many other plasma amino acids tended to be decreased by the exercise bout in control mice, but were not changed or rather increased by the exercise bout in BDK-mKO mice (S3 Fig); thus, the concentrations of alanine, arginine, aspartate, glutamate, glycine, histidine, lysine, and threonine tended to be higher in BDK-mKO mice than in control mice.

**Fig 3:**
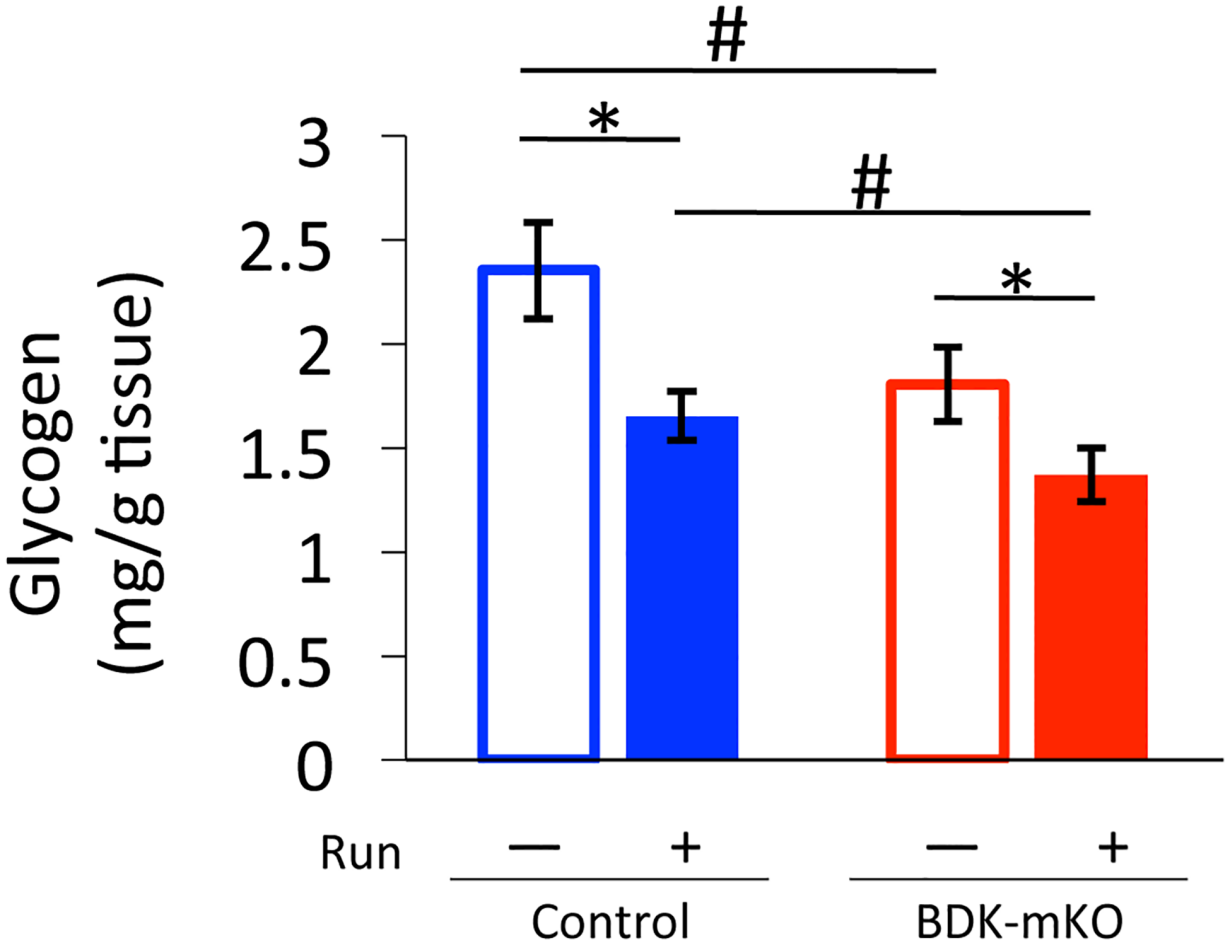
Glycogen contents in skeletal muscle of control and BDK-mKO mice with and without the exercise bout. # Significant difference between control and BDK-mKO mice. * Significant difference in the same group of mice with and without the exercise bout.

Since muscle glycogen content has been reported to be a factor affecting exercise performance [29], we determined the contents in the hind-limb muscle. Glycogen content was significantly lower in sedentary BDK-mKO mice than in sedentary control mice (Fig 3). The exercise bout significantly decreased the glycogen content in both groups of mice, resulting in lower glycogen content in BDK-mKO than in control mice after the exercise bout (Fig 3).

### Effects of BDK-mKO and exercise bout on the metabolome in skeletal muscle

#### BCAAs and their metabolites

**Fig 4:**
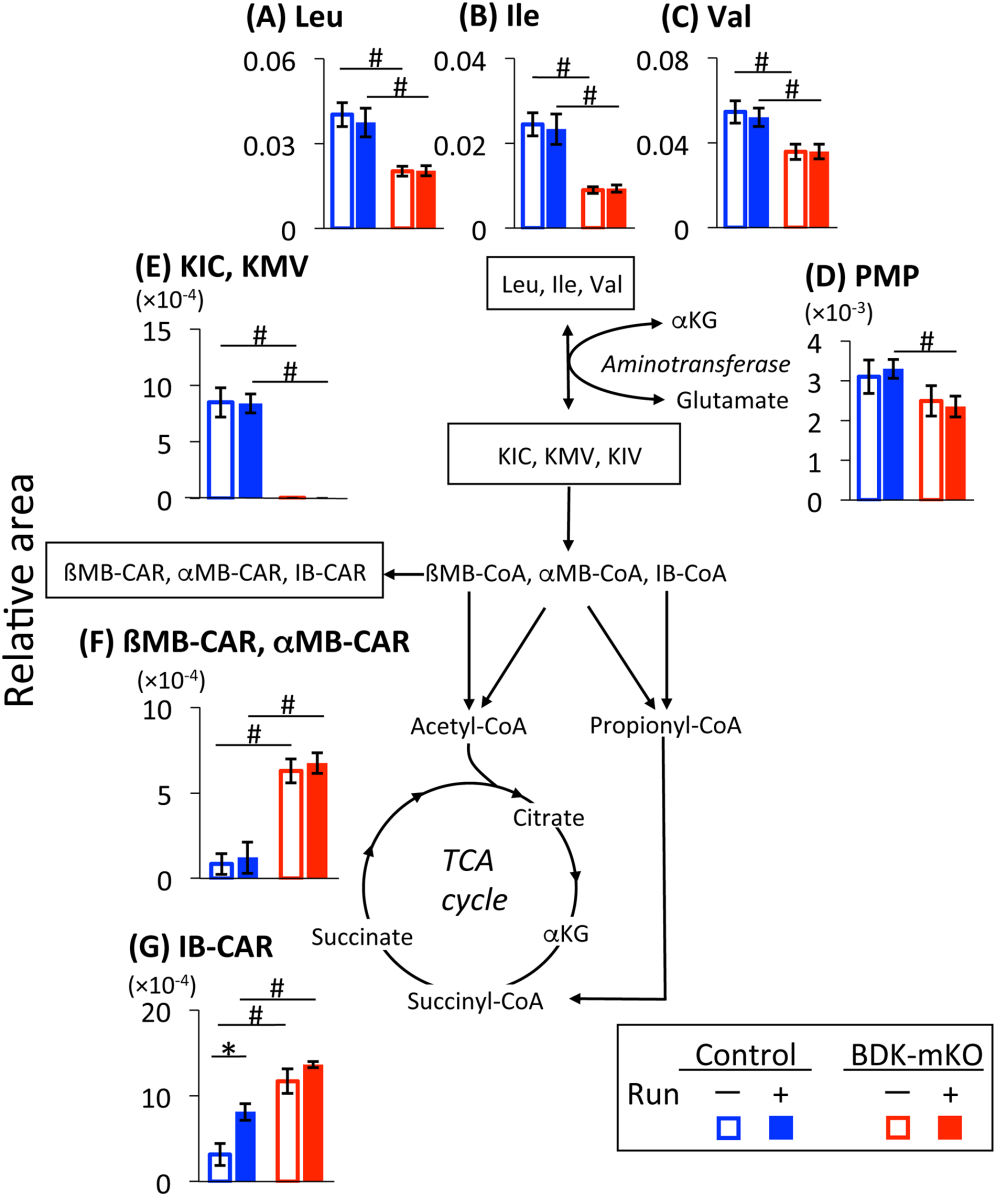
BCAAs and their metabolites. Changes in the metabolite levels in the skeletal muscle of control and BDK-mKO mice with and without the exercise bout are shown. # Significant difference between control and BDK-mKO mice. * Significant difference in the same group of mice with and without the exercise bout. PMP, pyridoxamine 5'-phosphate; KIC, α-ketoisocaproate; KMV, α-keto-ß-methylvalerate; KIV, α-ketoisovalerate; ßMB-CAR, ß-methylbutyryl-carnitine; αMB-CAR, α-methylbutyryl-carnitine; IB-CAR, isobutyryl-carnitine; and αKG, α-ketoglutarate.

The levels of the three BCAAs were significantly less in BDK-mKO mice than in control mice with and without the exercise bout; specifically, leucine and isoleucine levels were less than half in the former compared to the latter (Fig 4A–4C). These concentrations in both groups of mice were not affected by the exercise bout. The levels of alanine, aspartate, glutamate, histidine, lysine, and threonine tended to be higher in BDK-mKO mice than in control mice, but were not affected by the exercise bout (S4 Fig). Only the tryptophan level was increased by the exercise bout in both groups of mice.

The level of pyridoxamine 5-phosphate (PMP), a derivative of vitamin B6, tended to be lower in BDK-mKO mice than in control mice with and without the exercise bout (Fig 4D). α-Ketoisocaproic acid (KIC) and α-keto-ß-methylvaleric acid (KMV) were found at substantial levels in control mice, but were barely detected in BDK-mKO mice (Fig 4E). On the other hand, the levels of ß-methylbutyryl-carnitine (ßMB-CAR), α-methylbutyryl-carnitine (αMB-CAR) (Fig 4F) and isobutyryl-carnitine (IB-CAR) (Fig 4G) were significantly higher in BDK-mKO mice than in control mice. Only the IB-CAR level in control mice was affected by the exercise bout: the level was higher in control mice with exercise bout than in the same group of mice without exercise.

#### Metabolites in the glycolytic pathway

**Fig 5:**
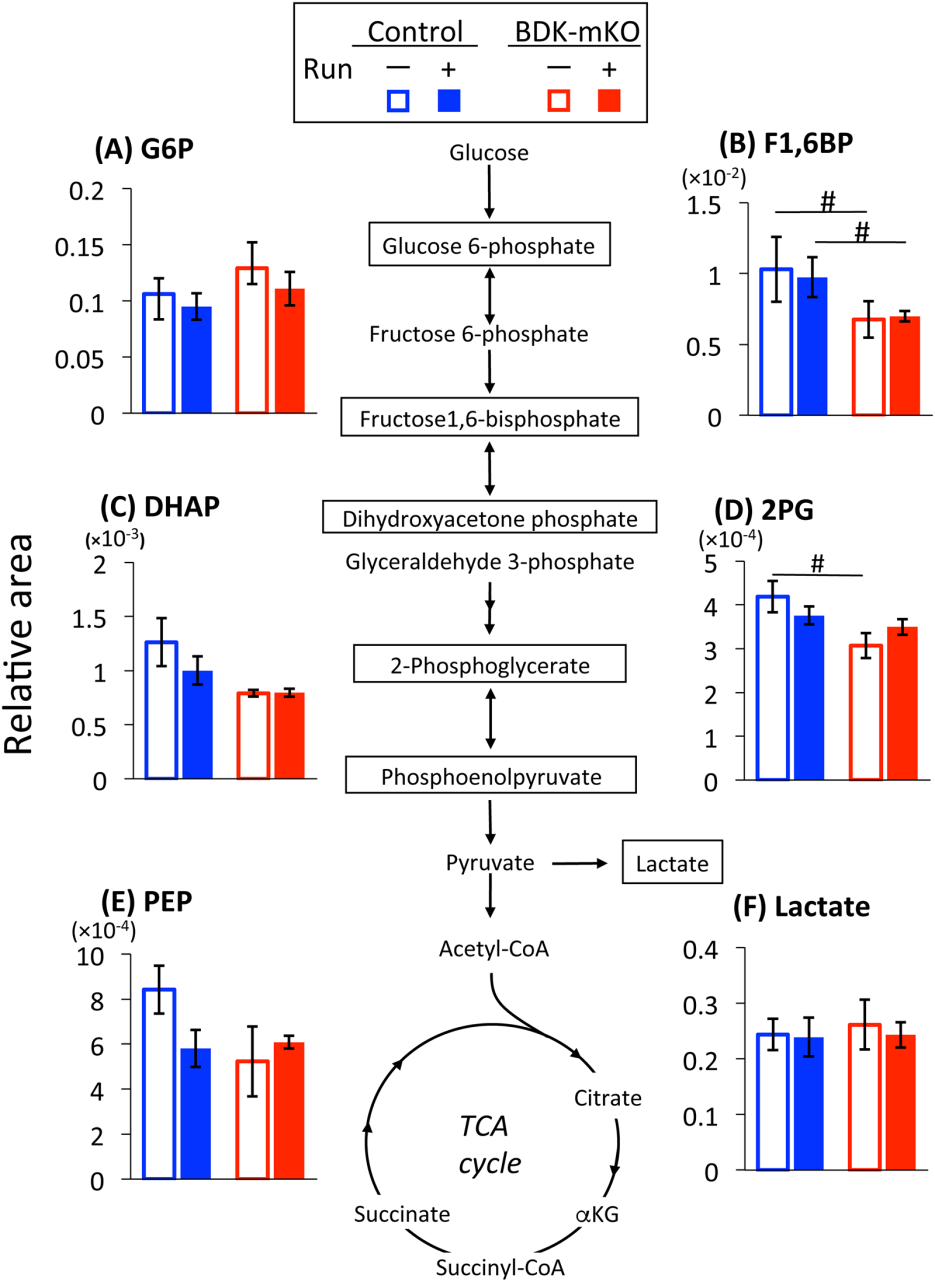
Metabolites in the glycolytic pathway. Changes in metabolite levels in skeletal muscle of BDK-mKO mice and control mice with and without the exercise bout are shown. # Significant difference between control and BDK-mKO mice. G6P, glucose 6-phosphate; F1,6BP, fructose 1,6-bisphosphate; DHAP, dihydroxyacetone phosphate; 2PG, 2-phosphoglycerate; and PEP, phosphoenolpyruvate.

There was no difference in glucose 6-phosphate (G6P) level in each group (Fig 5A). The levels of fructose 1,6-bisphosphate (F1,6BP) decreased in BDK-mKO mice with and without the exercise bout compared with control mice (Fig 5B). The levels of dihydroxyacetone phosphate (DHAP), 2-phosphoglycerate (2PG), and phosphoenolpyruvate (PEP) tended to be less in BDK-mKO mice than in control mice without the exercise bout (Fig 5C–5E), and these levels were largely not affected by the exercise bout. Muscle lactate also showed no difference in each group (Fig 5F).

#### Acetyl-CoA and metabolites in the tricarboxylic acid (TCA) cycle

**Fig 6:**
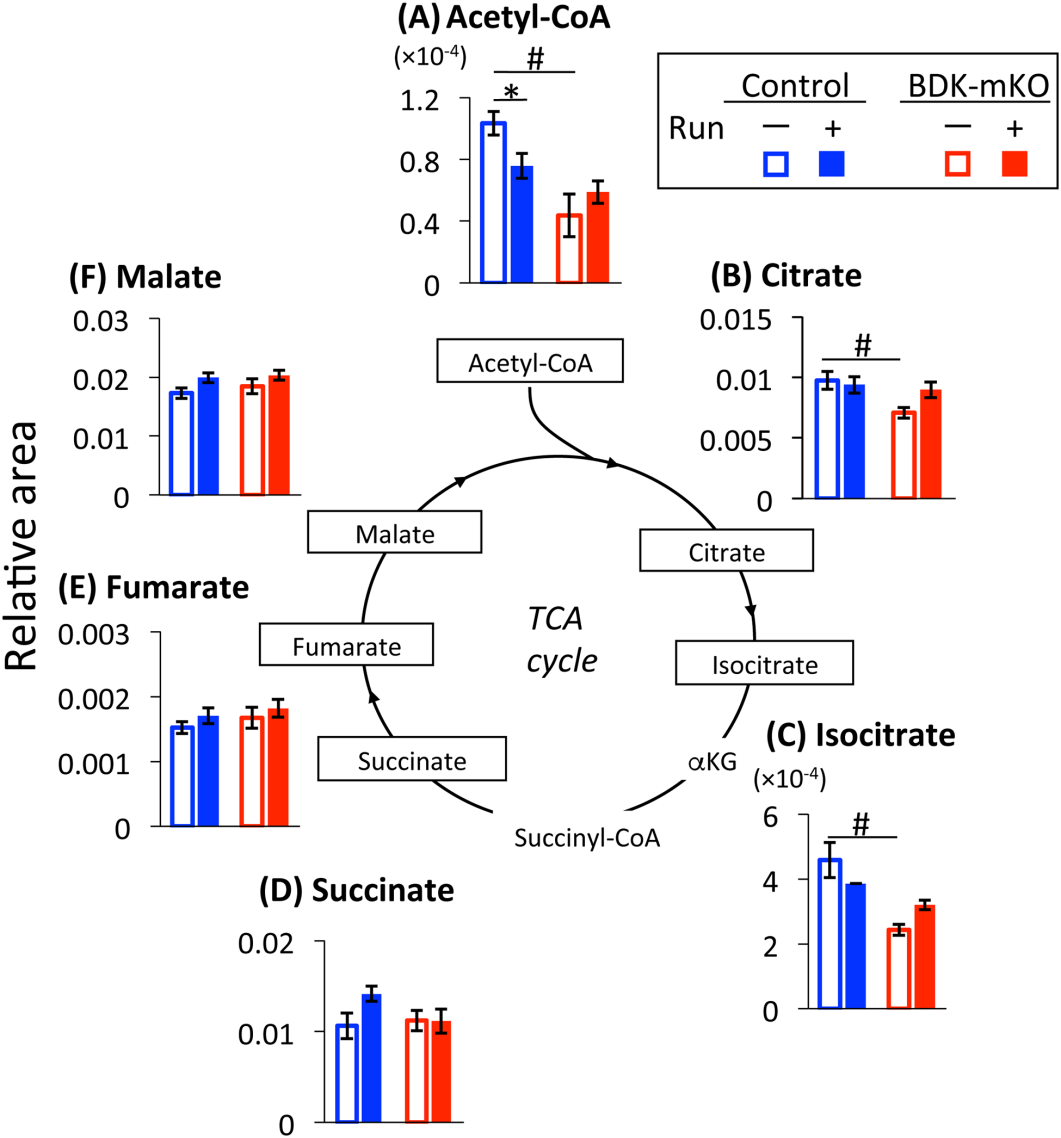
Acetyl-CoA and metabolites in the TCA cycle. Changes in metabolite levels in skeletal muscle of BDK-mKO mice and control mice with and without the exercise bout are shown. # Significant difference between control and BDK-mKO mice. * Significant difference in the same group of mice with and without exercise bout. αKG, α-ketoglutarate.

The levels of acetyl-CoA, citrate, and isocitrate were lower in BDK-mKO mice than in control mice without the exercise bout, and only the acetyl-CoA level in control mice was significantly decreased by the exercise bout (Fig 6A–6C). On the other hand, other metabolites (succinate, fumarate, or malate) in the cycle were not affected by BDK-mKO or the exercise bout (Fig 6D–6F).

#### NADH, NAD, and high-energy phosphate compounds

**Fig 7:**
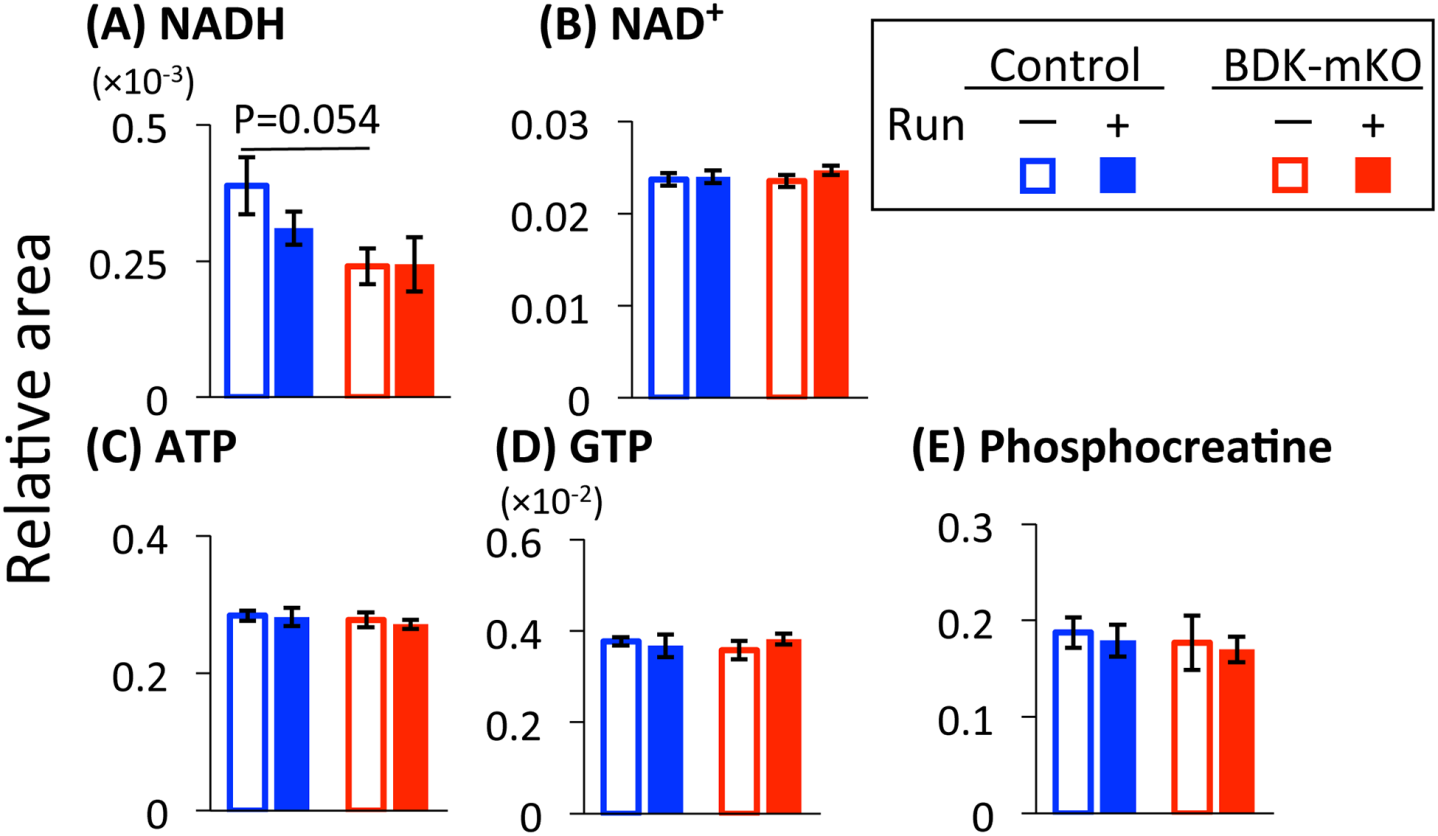
NADH, NAD^+^, and high energy compounds.

The NADH level tended to be lower in BDK-mKO mice than in control mice with and without the exercise bout, although the NAD^+^ level was the same among the 4 groups of mice (Fig 7A and 7B). The exercise bout tended to decrease the NADH level only in control mice. The levels of ATP, GTP, and phosphocreatine were the same between the control and BDK-mKO mice with and without the exercise bout (Fig 7C–7E).

### Mitochondrial enzyme activities

**Fig 8:**
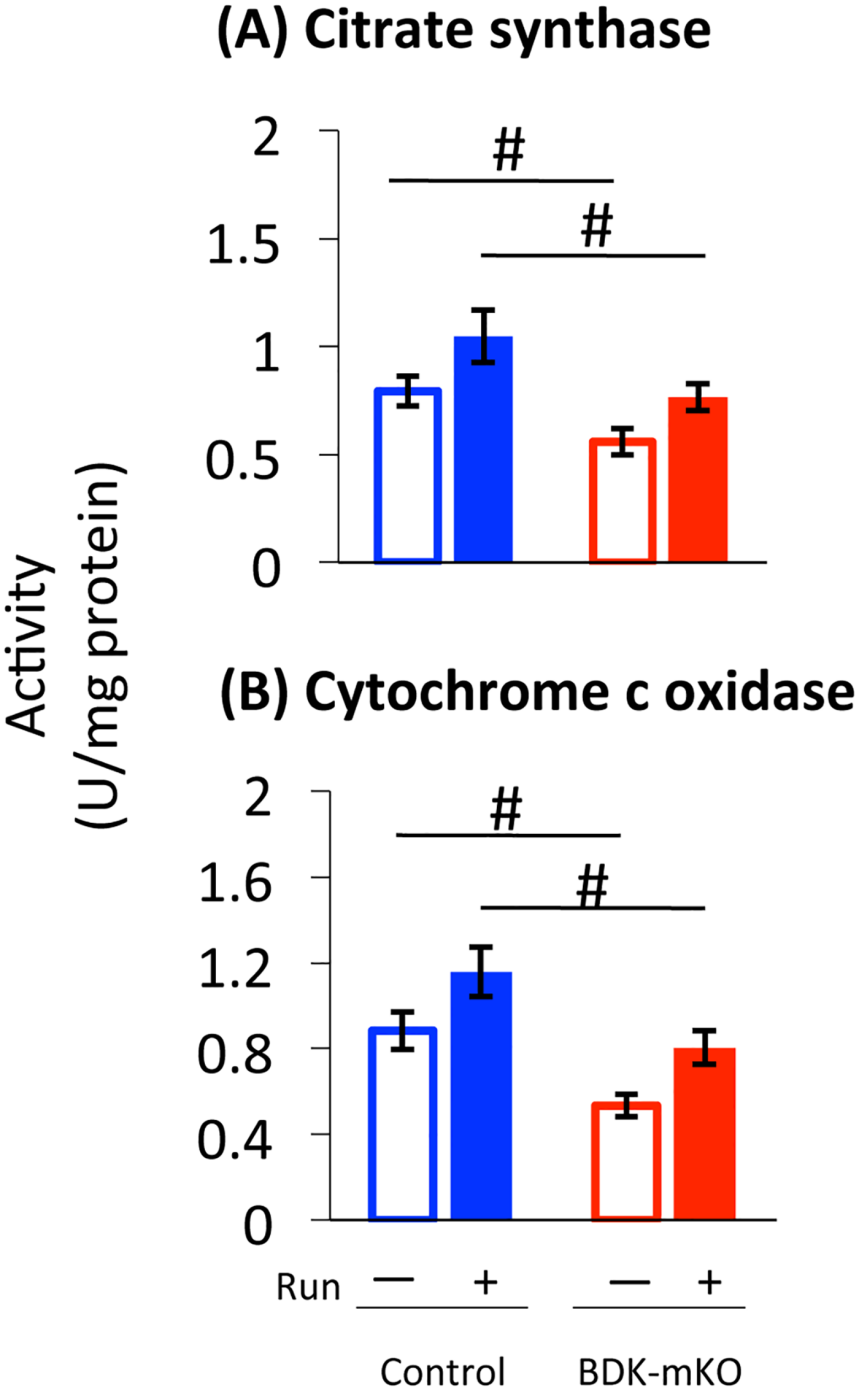
Citrate synthase and cytochrome c oxidase activities. # Significant difference between control and BDK-mKO mice.

We measured the activities of citrate synthase and cytochrome c oxidase as mitochondrial marker enzymes in soleus muscle, because this muscle is often used in the studies to examine effects of exercise on the mitochondrial enzyme activities. Both enzyme activities were less in BDK-mKO mice than in control mice with and without the exercise bout, although both enzyme activities tended to be increased by the exercise bout in both groups of mice (Fig 8).

## Discussion

BCAA catabolism in skeletal muscle is tightly regulated by BCKDC, most of which is inactivated by phosphorylation of the complex due to high BDK activity under normal and sedentary conditions [3, 9]. The low activity state of BCKDC suppresses BCAA oxidation, thereby contributing to the provision of BCAAs for muscle protein synthesis [30]. On the other hand, BCKDC in skeletal muscle is greatly activated by acute exercise in association with the decrease in the active form of BDK, which is bound to BCKDC [9], resulting in increased BCAA oxidation to supply substrates for enhanced muscle energy expenditure. In the present study, we demonstrated that the tight regulation of BCAA oxidation in muscle tissues is important, at lease in part, in the adaptation to exercise training; BDK-mKO mice in which muscle BCKDC was almost fully activated [17] showed somewhat, but significantly less adaptability to running and swimming exercise training. This down-regulation of exercise performance may be attributed to lower glycogen content and perturbation of energy metabolism. The decreased mitochondrial enzyme activities for energy production, which were induced by uncontrolled BCAA catabolism in soleus muscle, might be related to the exercise performance in BDK-mKO mice.

**Fig 9:**
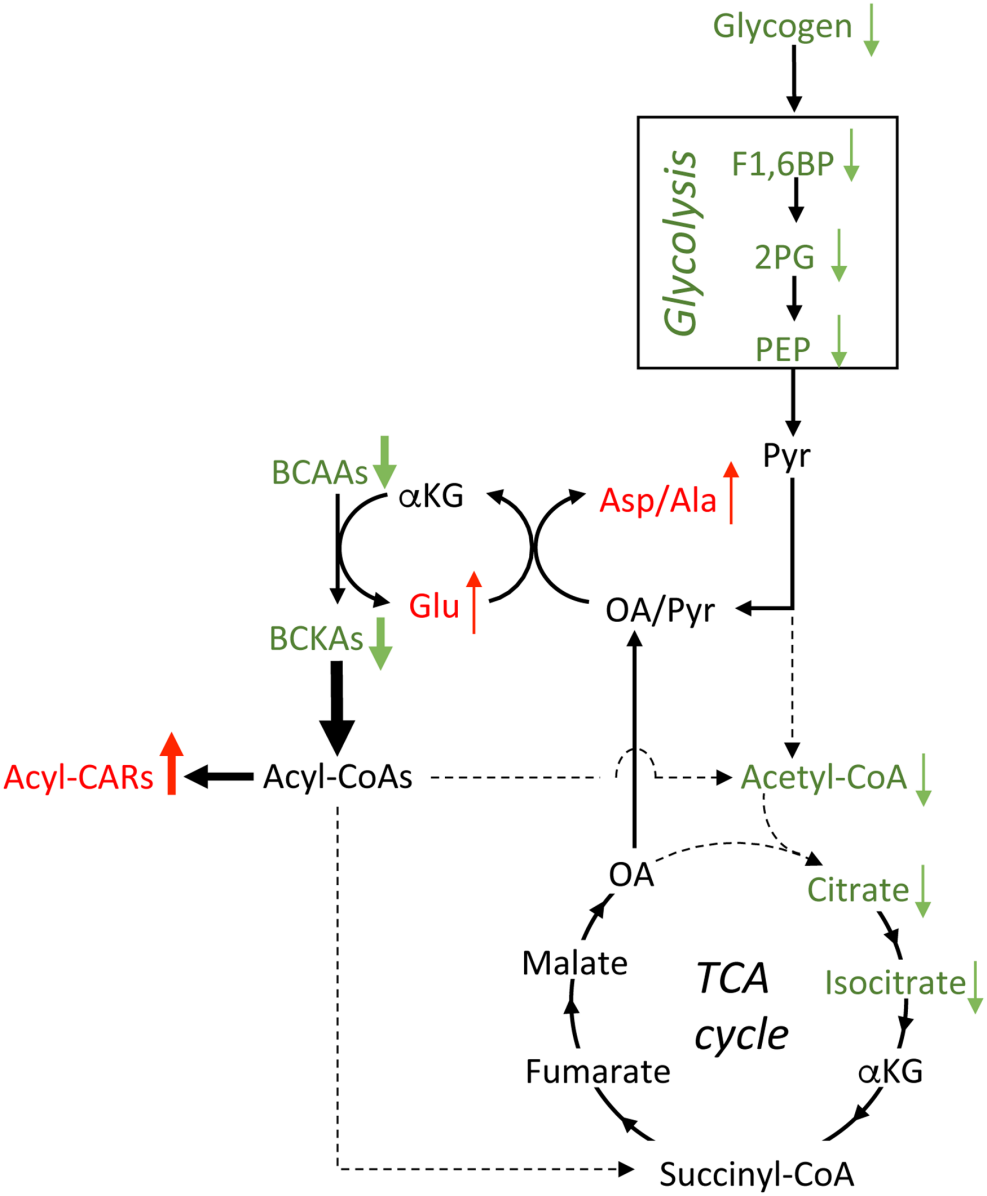
A schematic model of the perturbation of energy metabolism in association with enhanced BCAA catabolism. The low levels of muscle glycogen in BDK-mKO mice may lead to decreased levels of some metabolites in the glycolytic pathway. Accelerated decarboxylation of BCKAs promotes transamination of BCAAs, resulting in the production of Glu. The increased Glu may contribute to the production of Asp and Ala from oxaloacetate (OA) and pyruvate (Pyr), respectively, which may be responsible for the low levels of acetyl-CoA, citrate, and isocitrate in the muscle of BDK-mKO mice. On the other hand, accelerated decarboxylation of BCKAs produces acyl-CoAs, which appear to be converted mainly to acyl-carnitines (acyl-CARs). F1,6BP, fructose 1,6-bisphosphate; 2PG, 2-phosphoglycerate; PEP, phosphoenolpyruvate; and αKG, α-ketoglutarate.

From the metabolome analysis, BCAA levels were significantly decreased in association with dramatic decreases in the corresponding BCKAs and increases in the downstream metabolites, branched-chain acyl-carnitines, in skeletal muscles of BDK-mKO mice. These results indicate that catabolism of BCAAs and BCKAs was greatly enhanced in the muscles of BDK-mKO mice and largely produced the carnitine derivatives, but not acetyl-CoA or succinyl-CoA, in the muscles, suggesting that BCAA catabolism may not significantly contribute to energy metabolism. The levels of some components in the glycolytic pathway were downregulated in the muscles of BDK-mKO mice, which may be in line with the decrease in muscle glycogen content of the mice. These phenomena may be responsible for the low levels of acetyl-CoA in the muscles of BDK-mKO mice, presumably resulting in low levels of citrate and isocitrate in the TCA cycle and NADH. Since the perturbation of energy metabolism in the muscles of BDK-mKO mice was associated with the tendency for increased levels of alanine and aspartate in plasma and muscle tissues, it is thought that increased transamination of BCAAs produces glutamate from α-ketoglutarate, followed by the production of alanine and aspartate from pyruvate and oxaloacetate, respectively, by transamination (Fig 9). This hypothesis is supported by the evidence that, compared to oxaloacetate and pyruvate, α-ketoglutarate is the best amino group acceptor from BCAAs that is catalyzed by BCATm [31]. It should be noted that the metabolome analysis in the present study was conducted using mixture of gastrocnemius and plantaris muscles which are mainly composed of the anaerobic muscle fibers, because energy metabolism is significantly different between anaerobic and aerobic muscle fiber types. It has been reported that BDK-mKO had no effects on muscle fiber types of soleus and plantaris muscles [17].

In contrast to the low levels of acetyl-CoA and NADH in the muscles of BDK-mKO mice, levels of high energy compounds such as ATP were not affected by BDK-mKO, suggesting modest perturbation of energy metabolism by the accelerated BCAA transamination. The acute exercise bout used in this study could be classified as mild intensity, since all of the mice completed the running performance and few metabolites, such as acetyl-CoA, were decreased by the exercise bout and only in the control mice, although the muscle glycogen content was significantly decreased after exercise in both control and BDK-mKO mice.

We have reported that the tight regulation of BCAA catabolism is important in the homeostasis of muscle protein metabolism [17]. In the present study, we confirmed that this is also the case in energy metabolism of skeletal muscle. BCAAs are commonly used as supplements in the field of sports [32]. Although the BCKDC activity in liver was extremely higher in rat and mouse than humans, that in skeletal muscle is not so much different between rodents and humans [2]. Furthermore, it has been reported that BCAA oxidation is enhanced by exercise in association with an increase in the BCKDC activity in human and rat skeletal muscle [10, 11]. Therefore, the findings in the present study might be partly appricable to humans. However, further studies are required to elucidate the effects of excess BCAA supplementation, which may promote the catabolism of amino acids in humans.

## Supporting information

S1 Fig Western blotting of BDK and E2 component of the BCKDC in control and BDK-mKO mice. Tissue extracts were applied on SDS-PAGE, followed by transfer of proteins to PVDF membranes and immunostaining of BDK and E2 component, as described in Materials and Methods. Protein amounts per lane applied on the SDS-PAGE were 25 μg for heart, kidney, and pancreas; 30 μg for brain; and 50 μg for skeletal muscle, liver, spleen, and testis.
(PDF)

S2 Fig Typical Western blots of the BCKDC extracted from skeletal muscle of control and BDK-mKO mice. Age-matched untrained mice (both control and BDK-mKO mice) were prepared for this experiment. Therefore, trained and untrained mice (both control and BDK-mKO mice) were used in the Western blotting. The tissue extracts (gastrocnemius and plantaris muscles) were applied on SDS-PAGE, followed by transfer of proteins to PVDF membranes and immunostaining of BCKDC, as described in Materials and Methods. Protein amounts per lane applied on the SDS-PAGE were 25 μg.
(PDF)

S3 Fig Plasma concentrations of amino acids except for BCAAs in control and BDK-mKO mice with and without the exercise bout. # Significant difference between control and BDK-mKO mice. * Significant difference in the same group of mice with and without the exercise bout. Significant difference, p < 0.05.
(PDF)

S4 Fig Muscle levels of amino acids except for BCAAs in control and BDK-mKO mice with and without the exercise bout. # Significant difference between control and BDK-mKO mice. * Significant difference in the same group of mice with and without the exercise bout. Significant difference, p < 0.05.
(PDF)

S1 Table Body and tissue weights of control and BDK-mKO mice with and without the running exercise bout. Values are means ± SE. Skeletal muscle is a mixture of soleus, gastrocnemius, and plantaris muscles of the left hind-limb. Both sides of epididymal adipose tissues were collected from mice and combined.
(PDF)
