# Supplementary material for: Endurance performance and energy metabolism during exercise in mice with a muscle-specific defect in the control of branched-chain amino acid catabolism
Source: PLoS One. 2017 Jul 18;12(7):e0180989. doi: 10.1371/journal.pone.0180989 (PMC5515431; doi:10.1371/journal.pone.0180989)
Supplement: S1 Fig — Tissue extracts were applied on SDS-PAGE, followed by transfer of proteins to PVDF membranes and immunostaining of BDK and E2 component, as described in Materials and Methods. Protein amounts per lane applied on the SDS-PAGE were 25 μg for heart, kidney, and pancreas; 30 μg for brain; and 50 μg for skeletal muscle, liver, spleen, and testis. (PDF) [file pone.0180989.s001.pdf]

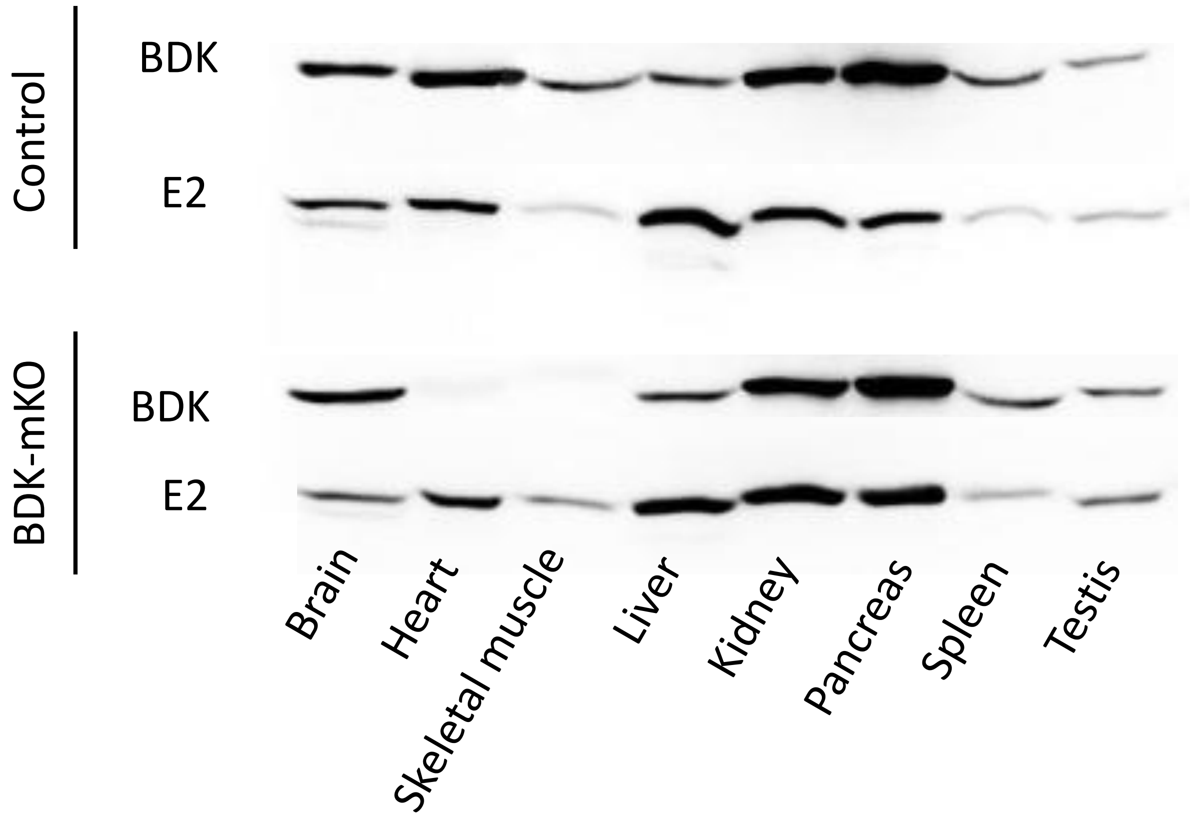

S1 Fig. Western blotting of BDK and E2 component of the BCKDC in control and BDK-mKO mice.

Tissue extracts were applied on SDS-PAGE, followed by transfer of proteins to PVDF membranes and immunostaining of BDK and E2 component, as described in Materials and Methods. Protein amounts per lane applied on the SDS-PAGE were 25 µg for heart, kidney, and pancreas; 30 µg for brain; and 50 µg for skeletal muscle, liver, spleen, and testis.
