# Supplementary material for: Endurance performance and energy metabolism during exercise in mice with a muscle-specific defect in the control of branched-chain amino acid catabolism
Source: PLoS One. 2017 Jul 18;12(7):e0180989. doi: 10.1371/journal.pone.0180989 (PMC5515431; doi:10.1371/journal.pone.0180989)
Supplement: S2 Fig — Age-matched untrained mice (both control and BDK-mKO mice) were prepared for this experiment. Therefore, trained and untrained mice (both control and BDK-mKO mice) were used in the Western blotting. The tissue extracts (gastrocnemius and plantaris muscles) were applied on SDS-PAGE, followed by transfer of proteins to PVDF membranes and immunostaining of BCKDC, as described in Materials and Methods. Protein amounts per lane applied on the SDS-PAGE were 25 μg. (PDF) [file pone.0180989.s002.pdf]

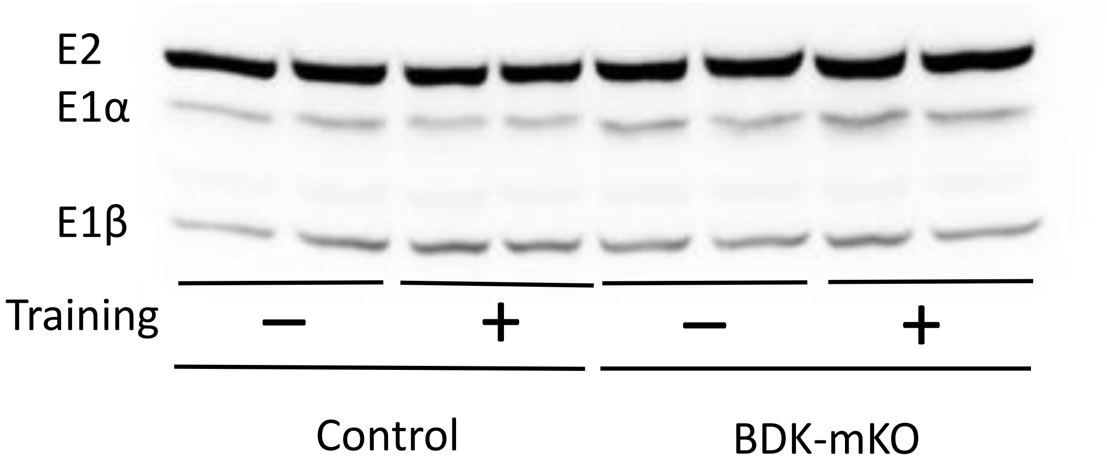

S2 Fig. Typical Western blots of the BCKDC extracted from skeletal muscle of control and BDK-mKO mice.

Age-matched untrained mice (both control and BDK-mKO mice) were prepared for this experiment. Therefore, trained and untrained mice (both control and BDK-mKO mice) were used in the Western blotting. The tissue extracts (gastrocnemius and plantaris muscles) were applied on SDS-PAGE, followed by transfer of proteins to PVDF membranes and immunostaining of BCKDC, as described in Materials and Methods. Protein amounts per lane applied on the SDS-PAGE were 25 µg.
