# Supplementary material for: Endurance performance and energy metabolism during exercise in mice with a muscle-specific defect in the control of branched-chain amino acid catabolism
Source: PLoS One. 2017 Jul 18;12(7):e0180989. doi: 10.1371/journal.pone.0180989 (PMC5515431; doi:10.1371/journal.pone.0180989)
Supplement: S4 Fig — # Significant difference between control and BDK-mKO mice. * Significant difference in the same group of mice with and without the exercise bout. Significant difference, p < 0.05. (PDF) [file pone.0180989.s004.pdf]

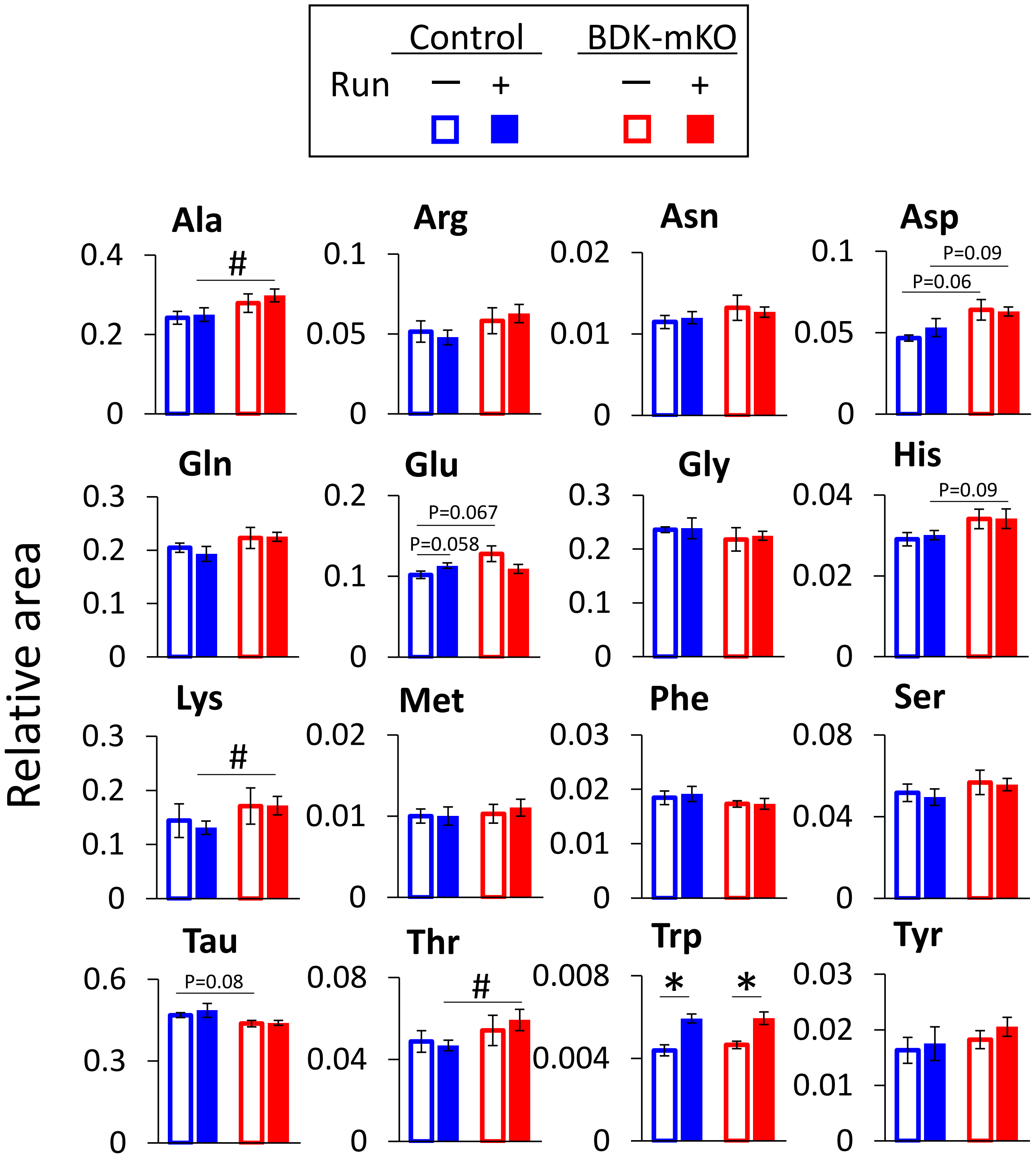

S4 Fig. Muscle levels of amino acids except for BCAAs in control and BDK-mKO mice with and without the exercise bout.
