# Supplementary material for: Endurance performance and energy metabolism during exercise in mice with a muscle-specific defect in the control of branched-chain amino acid catabolism
Source: PLoS One. 2017 Jul 18;12(7):e0180989. doi: 10.1371/journal.pone.0180989 (PMC5515431; doi:10.1371/journal.pone.0180989)
Supplement: S1 Table — Values are means ± SE. Skeletal muscle is a mixture of soleus, gastrocnemius, and plantaris muscles of the left hind-limb. Both sides of epididymal adipose tissues were collected from mice and combined. (PDF) [file pone.0180989.s005.pdf]

Supporting Information

S1 Table. Body and tissue weights of control and BDK-mKO mice with and without the running exercise bout

Item	Control		BDK-mKO	
	Run (–)	Run (+)	Run (–)	Run (+)
		(g)		
Body weight	27.5 ± 0.5	27.3 ± 0.7	25.9 ± 0.8	26.6 ± 0.7
Tissue		(g/100 g body weight)		
Skeletal muscle	0.52 ± 0.02	0.53 ± 0.03	0.55 ± 0.01	0.56 ± 0.01
Heart	0.44 ± 0.01	0.41 ± 0.02	0.47 ± 0.02	0.48 ± 0.01
Liver	4.10 ± 0.18	3.84 ± 0.16	4.02 ± 0.11	3.88 ± 0.07
Epididymal adipose tissue	1.92 ± 0.11	2.29 ± 0.11	1.63 ± 0.15	1.80 ± 0.14

Values are means ± SE. Skeletal muscle is a mixture of soleus, gastrocnemius, and plantaris muscles of the left hind-limb. Both sides of epididymal adipose tissues were collected from mice and combined.
